# Replacement of Congenitally Missing Maxillary Lateral Incisor with Two-Buccal-Retainer Resin-Bonded Fixed Dental Prosthesis Modified by Proximal Boxes

**DOI:** 10.1155/2022/5117542

**Published:** 2022-10-22

**Authors:** Lama Marmar, Safaa Bassam Shihabi, Issam Jamous

**Affiliations:** ^1^Department of Fixed Prosthesis, Faculty of Dentistry, Damascus University, Damascus, Syria; ^2^Department of Pediatric Dentistry, Faculty of Dentistry, Damascus University, Damascus, Syria

## Abstract

A 21-year-old male patient with congenitally missing maxillary right lateral incisor presented to Fixed Prosthodontics Department at the Faculty of Dentistry, Damascus University (Damascus, Syria). A two-buccal-retainer lithium disilicate glass-ceramic resin-bonded fixed dental prosthesis (RBFDP) was chosen to replace the missing tooth. This clinical report describes a novel preparation design of two-buccal-retainer lithium disilicate glass-ceramic RBFDP modified by proximal boxes. A recall appointment after 3 years showed no fracture, no de-bonding, no secondary caries, and no staining in prosthesis margins. Color matching with natural teeth was excellent, and the patient was satisfied.

## 1. Introduction

The restorative treatment for congenitally missing teeth in the anterior maxillary region is still a challenge in clinical dentistry and probably associated with loss of self-esteem and lower social status in young adults [1]. The main treatment options to replace missed anterior teeth are implant-supported prostheses [2], conventional fixed dental prostheses (FDPs) [3], and resin-bonded fixed dental prostheses (RBFDPs) [4].

Although an implant-supported prosthesis has become a common method to replace a missing tooth [5], there are still certain cases where implants are contraindicated. Psychiatric disorders, hematological malignancies, severe cardiovascular troubles, and patients receiving intravenous amino-bisphosphonates are general contraindications. A massive bone loss, absent of oral hygiene, and occlusal disorders are local contraindications [6]. In these cases, a tooth-supported prosthesis should be taken into consideration.

RBFDP is a minimally invasive tooth replacement option for a single missing anterior tooth, and it can be used as a definitive or interim prosthesis [1, 7].

RBFDPs are usually classified based on design (single-retainer or two-retainers) [4, 8, 9], the materials used (porcelain fused to metal or full ceramic) [10, 11], and tooth preparation type (palatal or buccal preparation) [1, 12].

Porcelain fused to metal RBFDPs has been introduced by Rochette [13], where perforated gold casting was used as a macro-mechanical bonding technique [14]. Almost a decade later, Maryland Bridge was developed, a non-perforated, nonprecious metal alloy framework with micro-mechanical retention using electrolytic etching [15].

Although these techniques are considered as minimally invasive, some disadvantages like frequent de-bonding, greying effect, and high abrasion rate were observed [7, 16]. With the increased evidence of the advantages of all-ceramic prostheses in both aesthetics and biocompatibility aspects, nowadays, numerous studies have shown the possibility to replace missing anterior teeth with all ceramic RBFDPs [17–19].

Different types of porcelain can be used to fabricate it, such as zirconia, glass-infiltrated alumina, feldspathic porcelain, and lithium disilicate glass-ceramic.

The microstructure of lithium disilicate glass-ceramic consists of 70% lithium disilicate crystals embedded in a glassy matrix [20]. This proportion has shown superior properties: high strength, high fracture resistance, and a high degree of translucency [21–23]. In addition, the glassy matrix makes the ceramic able to etch with hydrofluoric acid (HF), which gives this material the possibility to achieve a good micro-mechanical bonding and presents a relatively high bond strength [24].

Recently, clinical studies utilize lithium-disilicate glass ceramic RBFDPs with palatal or buccal preparation [12]. Buccal-retainer RBFDPs have superior aesthetic properties because of its capability in changing the color and shape of the abutment teeth [12]. However, studies showed that two-retainer lithium-disilicate glass ceramic RBFDPs still suffer from connector fracture [9, 24–26]. This clinical case report describes an innovative preparation design of lithium disilicate glass ceramic RBFDP with two-buccal-retainer for the replacement of congenitally missing maxillary lateral incisor.

## 2. Case Presentation

### 2.1. Diagnosis and Etiology

A 21-year-old healthy male patient with congenitally missing maxillary right lateral incisor was referred to Fixed Prosthodontics Department at the Faculty of Dentistry, Damascus University (Damascus, Syria). The unaccepted aesthetic appearance was the patient's chief complaint. Dental history showed that the patient received a fixed orthodontic therapy approximately one year before the first appointment. Then, a removable appliance with an artificial tooth was used to maintain the space and enhance the aesthetic appearance.

**Figure 1:**
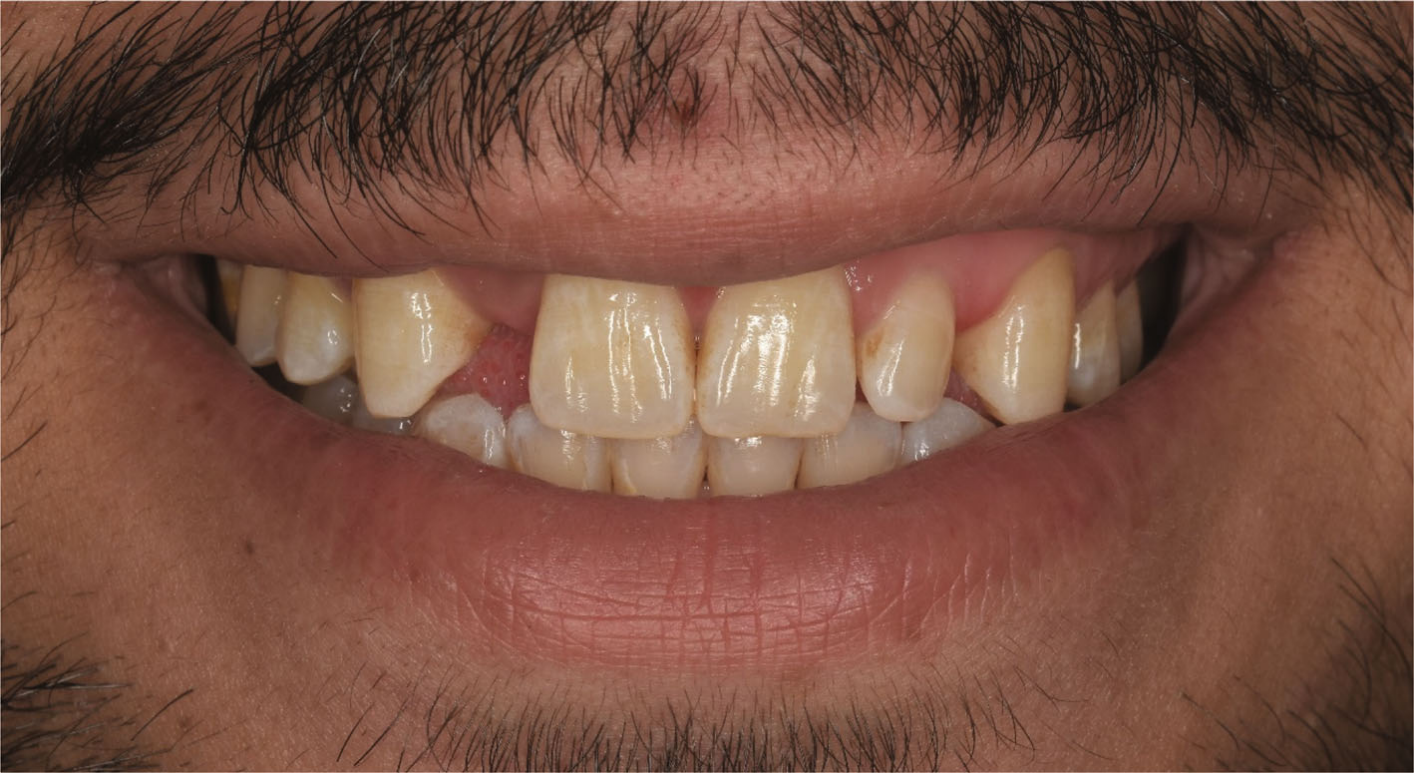
Smile view at initial evaluation.

The facial analysis appeared normal with no facial asymmetry. However, the smile analysis showed an asymmetry smile line (Figure 1).

**Figure 2:**
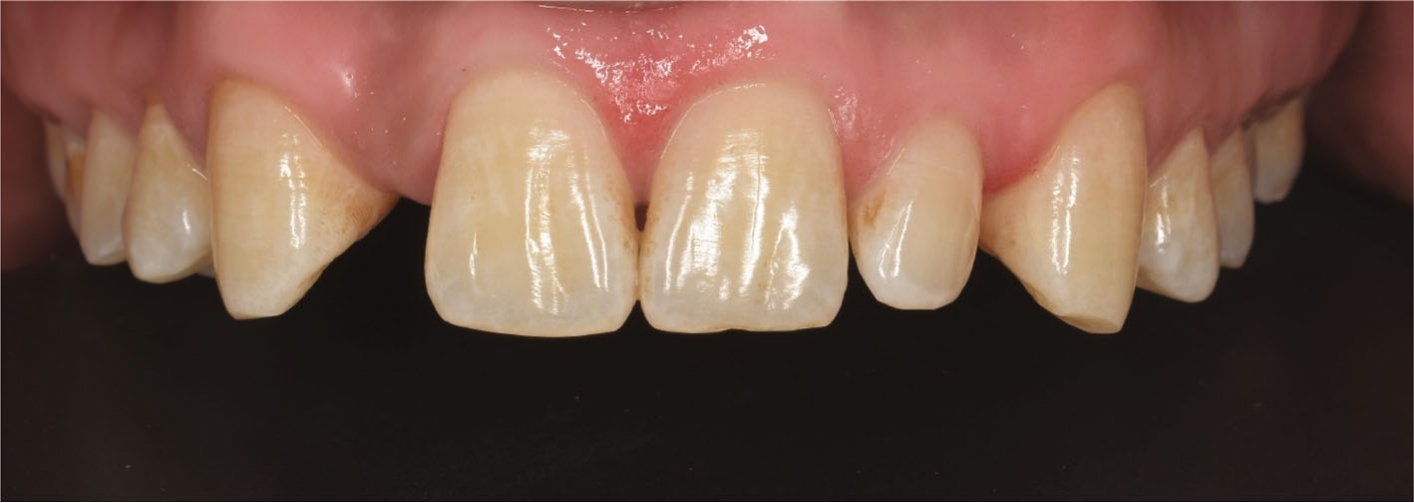
Pretreatment frontal view.

The dental examination showed that the maxillary central incisors and canines were intact. The left lateral incisor is smaller than normal. There is no symmetry between the right and the left incisors. The inter-coronal space for the maxillary right lateral incisor measured 4.5 mm. The molar and canine relationship was classified as class I according to angle classification of malocclusion, and the surrounding soft tissues were healthy (Figure 2).

The radiographic evaluation showed that the edentulous bone is inadequate for future implant placement in the maxillary right lateral incisor area.

### 2.2. Treatment Objectives

The treatment objectives were to replace the right lateral incisor and enhance the aesthetic appearance with conservative procedures.

### 2.3. Treatment Alternatives

The treatment alternatives were porcelain fused to metal or full ceramic conventional FDP, a single-buccal retainer RBFDP, and a single or palatal retainer porcelain fused to metal RBFDP.

### 2.4. Treatment Progress

Treatment options were discussed with the patient, and the approval of treatment plane was taken. Lithium-disilicate glass ceramic RBFDP with buccal retainers was selected as the treatment of choice to replace the right lateral incisor, and lithium-disilicate glass ceramic veneers was chosen to enhance the aesthetic appearance of left incisors.

**Figure 3:**
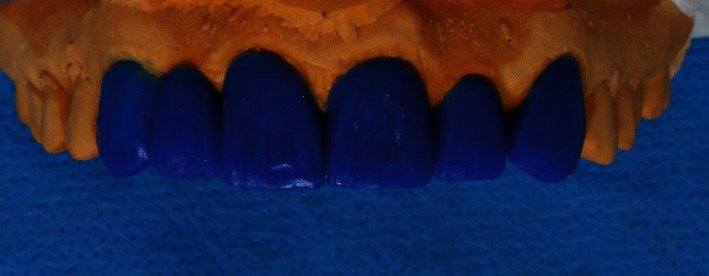
The wax-up model.

**Figure 4:**
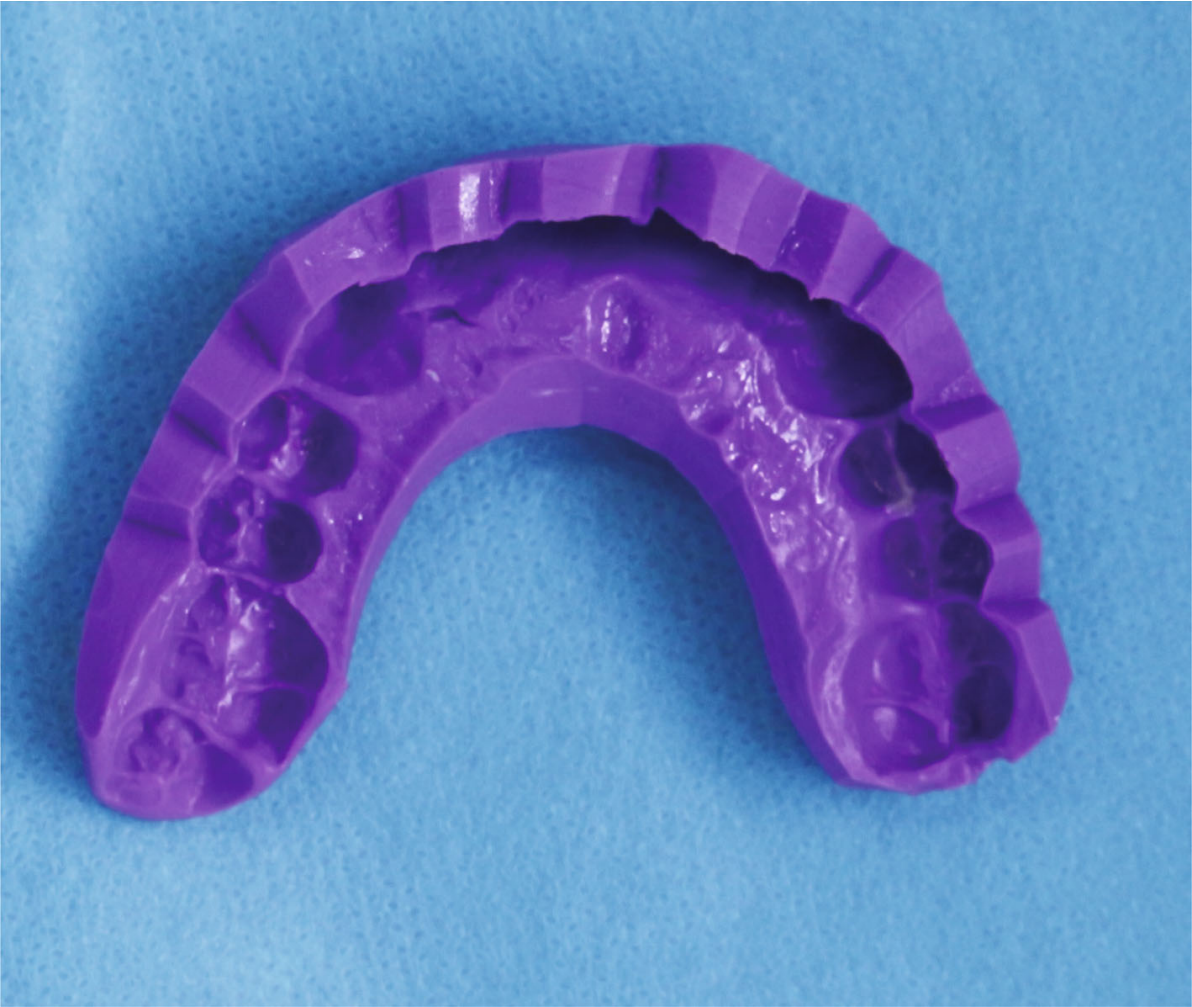
The silicone index.

Alginate impressions were taken, and diagnostic casts were made. The maxillary cast was used to make a wax-up model (Figure 3); then, it was used to make a silicone index, which was used to ensure appropriate depth of the preparation and make the provisional FDP (Putty and Light Body, tg, Germany; Figure 4).

The abutments were prepared according to the general guidelines of laminate veneer preparation, taking the path of insertion of FDPs into consideration.

**Figure 5:**
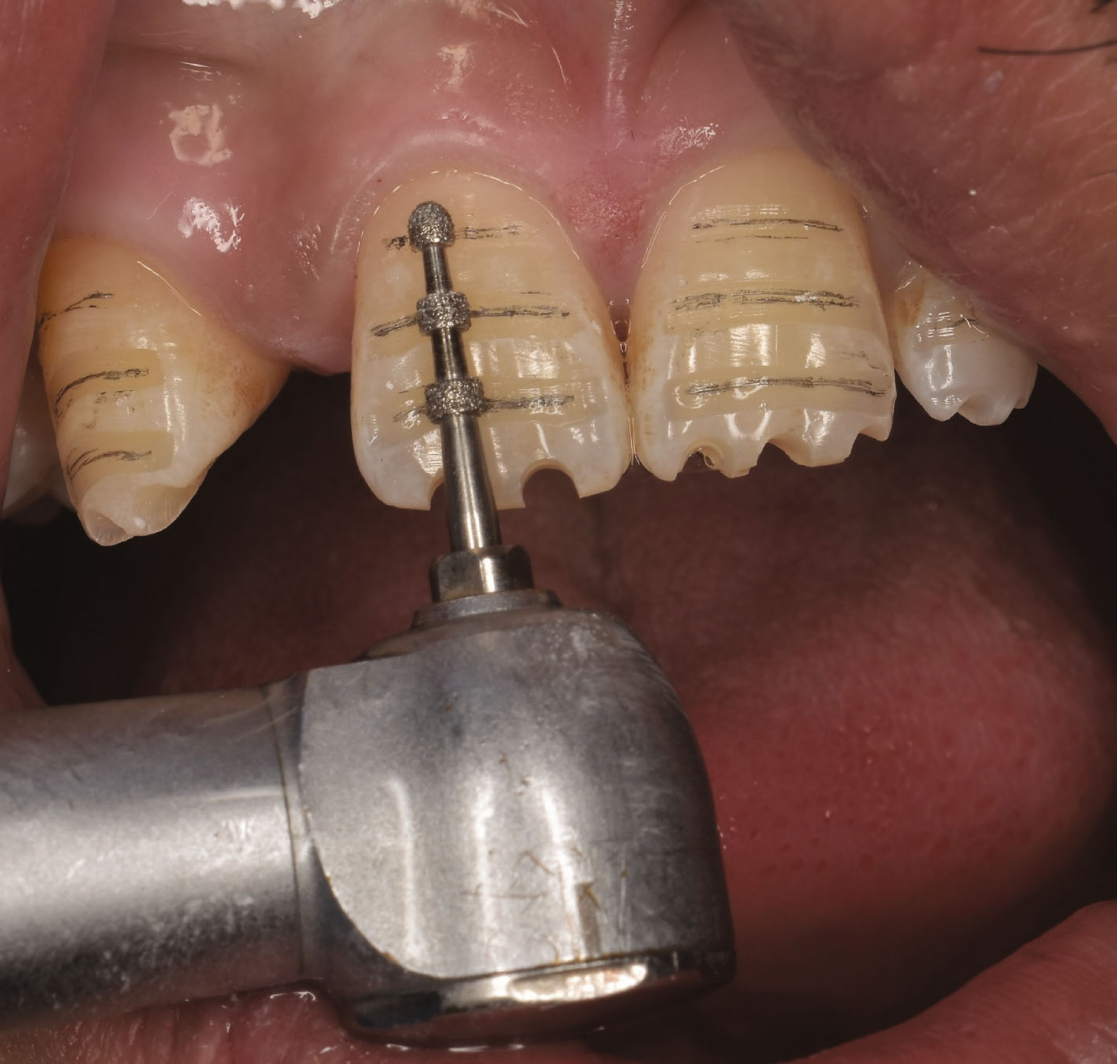
Depth marking bur.

The incisal index grooves were done using a cylinder diamond bur (Torpedo Diamond Bur, Komet Dental, Gebr. Brasseler, Lemgo, Germany), and the buccal preparation depth was determined using a depth-marking bur (#834-016, Komet Dental, Gebr. Brasseler; Figure 5).

**Figure 6:**
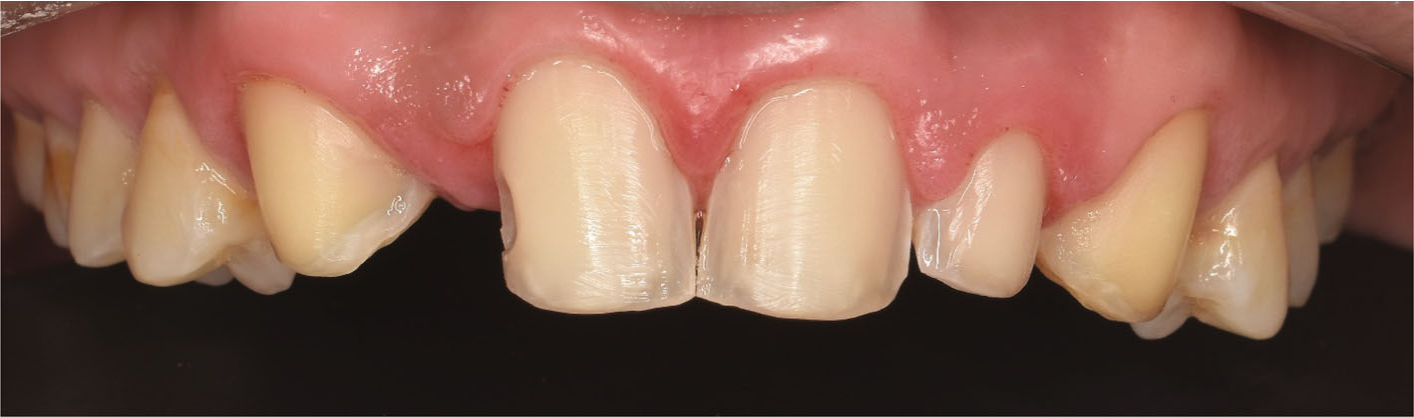
Frontal view of abutment tooth preparations.

The incisal reduction was 1.5 mm, and the incisal preparation design was butt joint. The buccal preparation was made using a tapered round-end diamond bur (#868-314-016, Komet Dental, Gebr. Brasseler), in order to provide a thickness of 0.7–1 mm for the retainers and 0.5 mm chamfer finish line (Figure 6).

**Figure 7:**
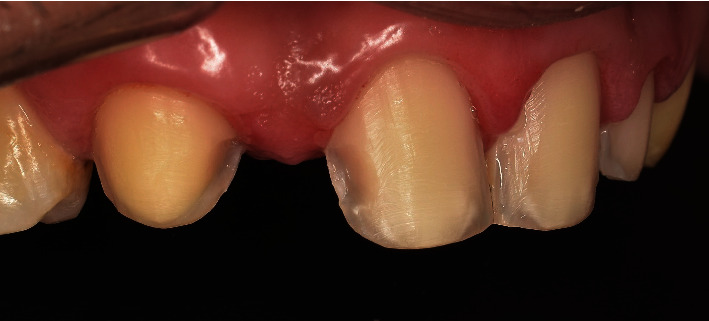
Buccal preparation design (proximal boxes).

The proximal preparation was extended to the proximo-palatal line angles adjacent to the edentulous area with 0.5 mm chamfer finish line. Our new strategy was to prepare a proximal box in the pontic side of each abutment using a cylinder round-end diamond bur (Torpedo Diamond Bur, Komet Dental, Gebr. Brasseler). These boxes measured 2 mm bucco-palatal dimension, 2 mm cervical-incisal dimension, and just 0.5 mm axial dimension. The box should be placed 2 mm far from the gingiva and 1 mm far from the incisal edge. The two proximal boxes should be parallel and diverge to the buccal surface (Figure 7).

Fine diamond burs (#8868-314, Komet Dental, Gebr. Brasseler) were used to round all internal line angles and finish all surfaces.

**Figure 8:**
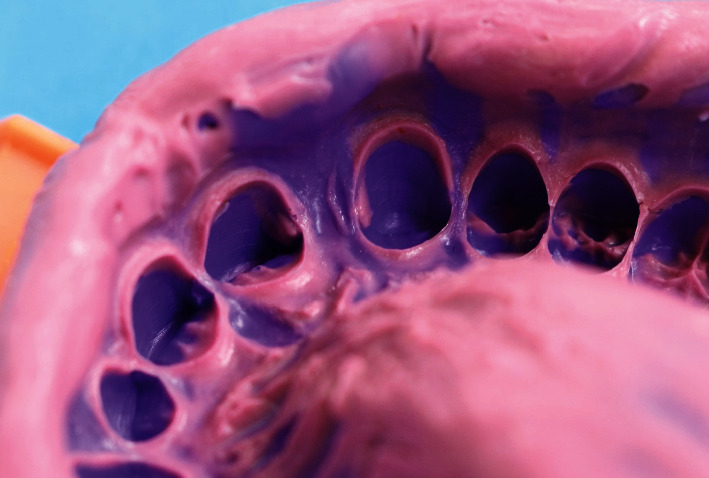
Putty wash impression.

A full arch addition silicone impression (Putty and Light Body) was done using a putty wash impression technique (Figure 8).

**Figure 9:**
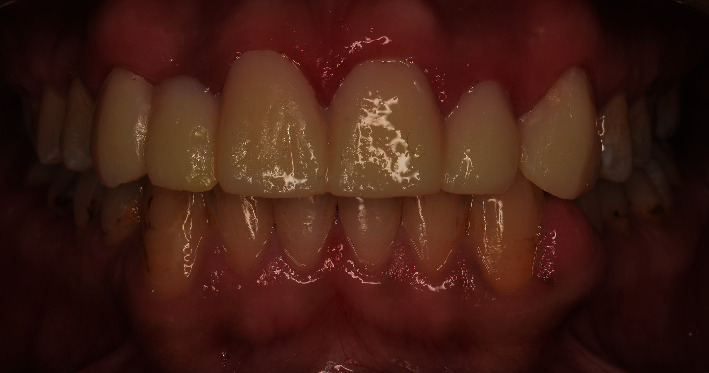
The provisional FDP.

Based on the primary wax-up model, a provisional FDP was made of auto-polymerizing resin material (Provi Temp K, Bisico, Germany); then, it was cemented with a flowable light polymerizing composite (Tetric N-Flow, Ivoclar Vivadent; Figure 9).

**Figure 10:**
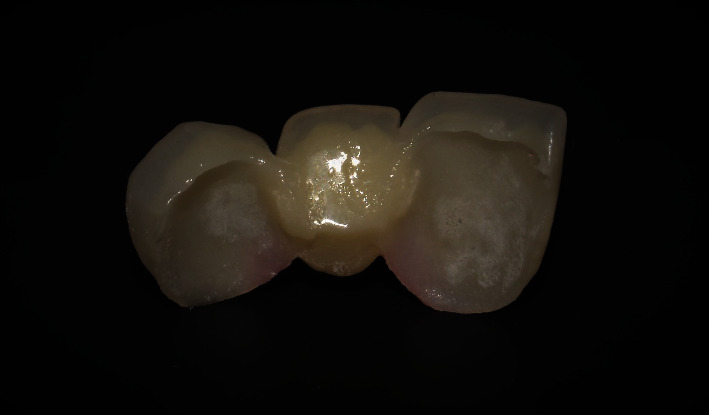
Definitive IPS E.max Press based RBFPDs.

A full contour lithium disilicate glass ceramic RBFDP with two-buccal-retainer was heat-pressed using a low-translucency ceramic ingot (Low Translucency A2 ingot, IPS E.max Press, Ivoclar Vivadent; Figure 10). The final shade was obtained by applying external stains (IPS E.max Ceramic Shades, Essence, Ivoclar Vivadent). The modified ridge lap design was chosen for the pontic.

At the try-in appointment, complete seating and marginal adaptation of the prosthesis were evaluated; static and functional occlusions were adjusted; the aesthetic properties, shape and color matching were assessed. The final approval of the patient was obtained.

**Figure 11:**
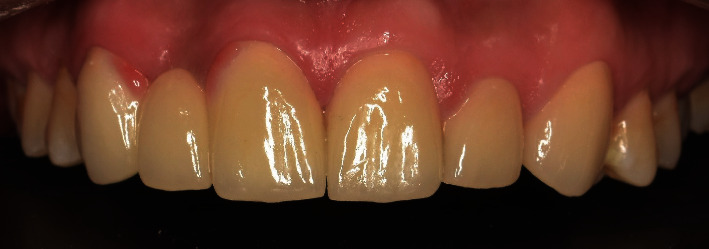
Postoperative frontal views of prosthesis.

The prosthesis was cemented with A1 shade light polymerizing resin cement (Variolink N, Base, Ivoclar Vivadent) according to the manufacturer's instructions (Ivoclar Vivadent). The internal surfaces of the prosthesis were acid etched with HF 5% (IPS Ceramic etching gel, Ivoclar Vivadent) for 20 seconds [27]; then, it was rinsed with water spray and dried with oil-free air. The etched surfaces were treated with a silane-coupling agent (Monobond plus, Ivoclar Vivadent). The enamel was etched with 37% phosphoric acid (N-Etch, Ivoclar Vivadent) for 30 seconds. A bonding agent (Excite F, Ivoclar Vivadent) was applied to all bonding surfaces of the abutments. The light cure resin cement was applied directly to the internal surfaces of the treated prosthesis, and the prosthesis was fixed (Figure 11). The excess cement was removed with a micro-brush, and the light cure was applied. The static and functional occlusions were evaluated with 80 *μ* articulating paper (Articulating paper, Corta, Germany). Fine diamond burs and porcelain polishing kit (Optrafine, Ivoclar Vivadent) were used to make the occlusal adjustments.

**Figure 12:**
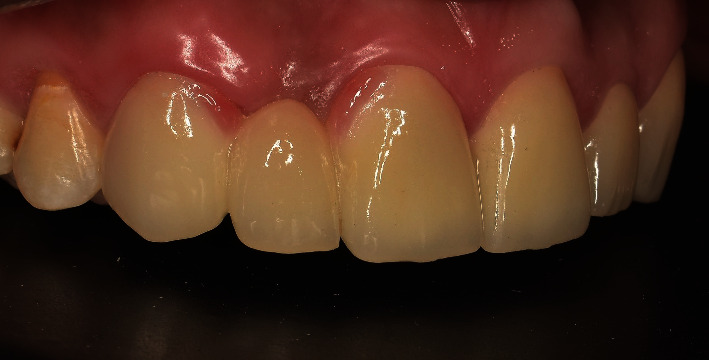
Recall after 3 years.

**Figure 13:**
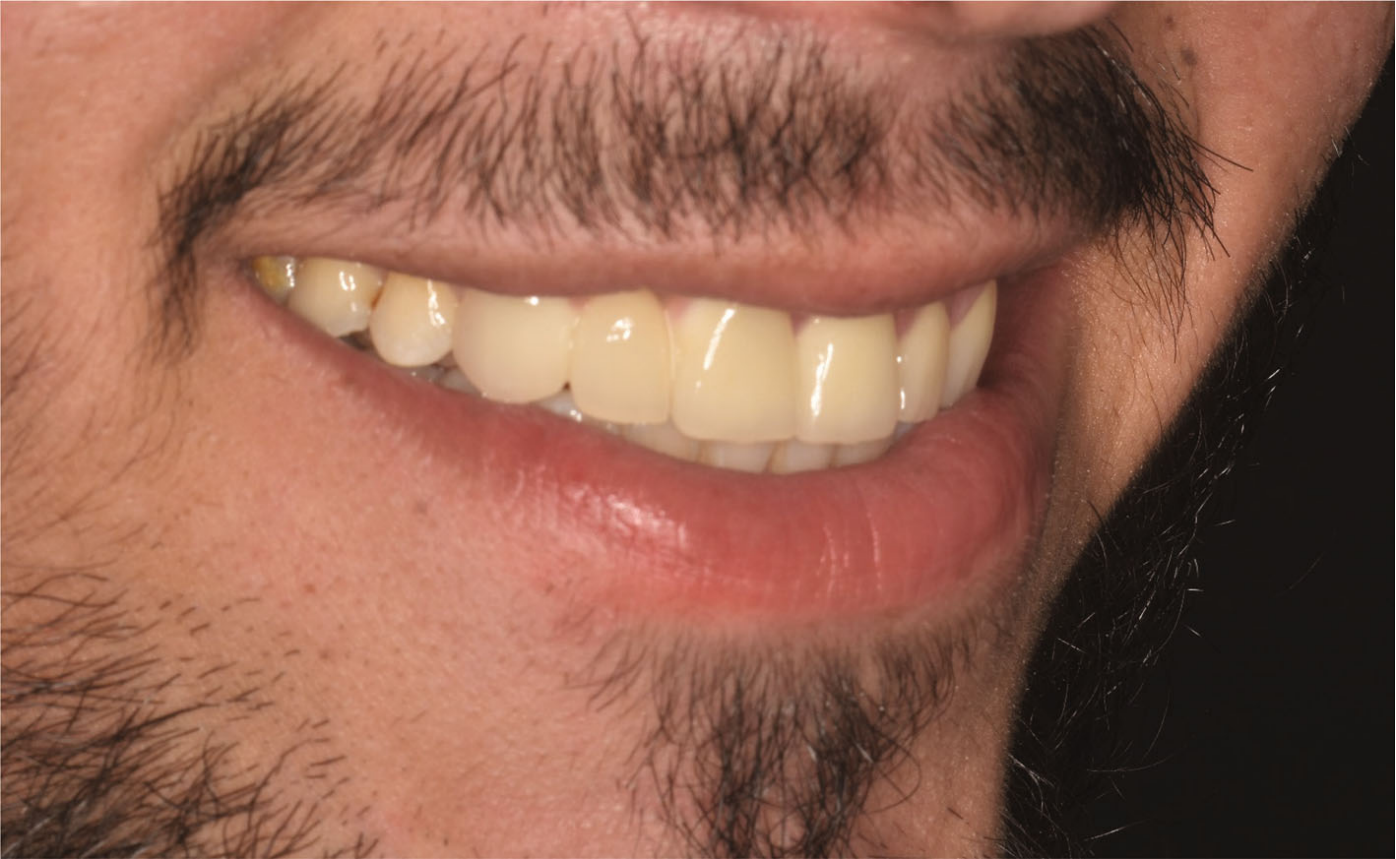
A smile view.

Recall appointments were performed 8 times over a 3-year period (Figure 12). No fracture, no de-bonding, no secondary caries, and no staining in the margins of retainers were observed. Color matching with natural teeth was excellent, and the patient was satisfied (Figure 13).

## 3. Discussion

Different therapeutic options can be indicated for replacement of missing lateral incisor. Although implants are often the most popular treatment choice [6], in this case, the inter-coronal space between the two abutments was insufficient for an implant even after the achieved orthodontic treatment, which caused decreasing the width and the high of the alveolar bone as a result of moving the canine distally and the central incisor mesially [28]. Therefore, in our case, replacing the lateral incisor with implant requires applying another orthodontic appliance, then grafting and implantation, which conflict with the patient's financial background. The conventional FDP is an aggressive treatment choice for such an intact abutments; it needs a full crown preparation, which removes around 72% of the abutment's weight [29]. Therefore, a more economic and conservative treatment should be chosen.

RBFDP is a cost-effective replacement treatment, the preparation does not remove more than 30% of the abutments' weight, and it can be used as a definitive prosthesis [7, 29]. In this case, the abutments need shape modifications, so buccal-retainer RBFDP was elected to change the abutment shape and enhance the color-matching taking into consideration that the translucency and strong mechanical properties are the most important factors that should be evaluated when choosing the restorative materials. As the mastication force peaks are not more than 200 N in anterior region, lithium disilicate glass-ceramic was chosen to fabricate the prostheses because of its high aesthetic properties and its ability to undertake a load up to 500 N [25, 30, 31].

For these reasons, the two-buccal-retainer lithium disilicate glass-ceramic RBFDP was chosen to replace the missing tooth.

The abutments prepared according to the general guidelines of a porcelain veneers preparation and the path of insertion of the FDP were taken into consideration. The proximal preparation was extended to the proximo-palatal line angle adjacent to the edentulous area to provide an adequate bucco-palatal dimension for the connectors [1, 12]. The proximal boxes were prepared to get additional thickness of ceramic in connecter area, which provide more fracture resistance and high survival rate for two-buccal-retainer lithium disilicate glass-ceramic RBFDP [32]. The axial dimension of the boxes was just 0.5 mm to keep the preparation within the enamel thickness, which is approximately 1.0 mm in the proximal area [33]. The butt joint preparation design was chosen for the incisal edges to make the path of insertion bacco-palatally as the path of the proximal boxes. The full contour hot press technique was chosen to fabricate the bridge as the pressed prostheses have shown better marginal fit compared with CAD CAM ones [34, 35], and the full anatomic prostheses have superior fracture resistance compared with those made by bi-layered technique [36]. Modified ridge lap design was chosen for the pontic as studies recommended for anterior maxilla because it combines aesthetics, biocompatibility, function, phonetics, patient comfort, and maintenance of healthy soft tissue [37]. The cementation was done using light cure resin cement following the most accepted surface treatment protocol for enamel and lithium disilicate glass-ceramic, which includes etching with 5% HF followed by a silane coupling agent for lithium disilicate glass-ceramic and etching with 37% phosphoric acid followed by bonding agent for the enamel [38, 39]. No enamel pretreatment was done before etching. However, a new study suggests enamel pretreatment with erythritol or sodium bicarbonate to enhance the bonding strength [40]. Therefore, further studies should be conducted to evaluate this protocol in order to improve the successful rate of this prosthesis. Two-buccal-retainer lithium disilicate glass-ceramic RBFDPs were utilized successfully in the patient with congenitally missing maxillary lateral incisor due to appropriate case selection, and the proximal boxes modified preparation technique.

## 4. Conclusion

Over 3-year follow-up recall, neither de-bonding nor fracture was observed. No secondary carries and no staining in the retainers' margins had been found. The color matching with the adjacent natural teeth was excellent, and the patient was satisfied. More studies with longer follow-up are needed to confirm the result of this novel design of anterior RBFDPs.
